# The effect of wind speed in increasing COVID-19 cases in Jakarta: a spatial-temporal analysis from March to September 2020

**DOI:** 10.12688/f1000research.128908.3

**Published:** 2023-12-28

**Authors:** Dewi Susanna, Yoerdy Agusmal Saputra, Sandeep Poddar

**Affiliations:** 1 Department of Environmental Health, Faculty of Public Health, Universitas Indonesia, Depok, Jawa Barat, 16424, Indonesia; 2 Study Program of Public Health, Institut Kesehatan Indonesia, Jakarta Utara, Jakarta, 14240, Indonesia; 3 Research and Innovation, Lincoln University College, Petaling Jaya, Selangor, 47301, Malaysia

**Keywords:** COVID-19, SAR-CoV-2, maximum wind speed, average wind speed, spatial–temporal analysis

## Abstract

**Background:**

The SARS-CoV-2 virus that causes COVID-19 is described as a highly contagious virus, and wind speed is suspected to be one of the climate elements that play a role in its spread, among others. This study aims to determine the relationship between wind speed and the increase in COVID-19 cases, as well as its potential spread, based on regional characteristics.

**Methods:**

The design of this study was an ecological study based on time and place to integrate geographic information systems and tested using statistical techniques. The data used were wind speed and weekly COVID-19 cases from March to September 2020. These records were obtained from the special coronavirus website of Jakarta Provincial Health Office and the Indonesian Meteorology, Climatology and Geophysics Agency. The data were analyzed by correlation, graphic/time trend, and spatial analysis.

**Results:**

The wind speed (maximum and mean) from March to September 2020 tended to fluctuate between 1.43 and 6.07 m/s. The correlation test results between the average wind speed and COVID-19 cases in Jakarta showed a strong positive correlation (r = 0.542; p value = 0.002).

**Conclusions:**

Areas with high wind speeds tended to show an increase in the number of COVID-19 cases, especially in the coastal areas of Jakarta. Wind speed plays a role in increasing the spread of SARS-CoV-2, in people who did not implement health protocols properly. This mechanism can be worsened with support of environmental factors such as air pollution.

## Introduction

Several nations are currently experiencing a significant increase in coronavirus (COVID-19) cases, including Indonesia ( https://worldometers.info/coronavirus/). A total of 34,874,744 confirmed cases with 1,097,497 deaths (case fatality rate (CFR) 3.1%) were reported in 216 countries based on data from the World Health Organization ( https://www.who.int/docs/default-source/coronaviruse/situation-reports/20200831-weekly-epi-update-3.pdf?sfvrsn=d7032a2a_4). In Indonesia, the number of people who have been infected and the number who have died are approximately 287,008 and 10,740 (CFR 3.7%), respectively, with the most predominant regions being Jakarta (73,700), East Java (43,536), and Central Java (22,440) ( https://covid19.go.id/peta-sebaran).

Based on various studies worldwide, the SARS-CoV-2 virus responsible for COVID-19 is described as highly contagious. ^1^ Overall, the transmission dynamics of viral infections, such as COVID-19, are caused by systemic factors (biological characteristics of the virus, incubation period, etc.), specific factors (complex interactions between air pollution, meteorological conditions, etc.), biological characteristics of viral infectivity, and the health level of the individual (habits, immune system, age, gender, etc.). ^2^

Saliva droplets produced by asymptomatic carriers appear to be the possible transmission media. In addition, specific observations using highly sensitive laser beams indicated that loud speech tends to emit countless droplets of oral fluid per second. In a closed and stagnant air environment, the drops finally disappear from the viewing window within the range of eight to14 minutes. Based on these findings, regular speech shows a high probability of transmitting the airborne virus in confined spaces. ^1^ Therefore, aerosol transfer appears to be the most significant method of SARS-CoV-2 spread compared with other media. ^1^^,^^3^^,^^4^

The role of climatic elements such as temperature, humidity, rainfall, wind speed, and sunlight is very important in disease transmission because these are parameters that can determine the survival of the SARS virus in the environment. ^5^^–^^9^ Recent studies have shown that the virus remains active in airborne particles after three hours. ^10^^,^^11^ In addition, these particles also settle on various surfaces and can attach to air pollutants if weather conditions are stable and particulate matter (PM) concentrations are high. ^12^^,^^13^ High wind speeds can accelerate the spread of the virus by carrying droplets to various destinations. ^14^^–^^19^

The increase in temperature caused by hot and sunny weather can increase air circulation in the environment, which results in a decrease in air pollution, reducing the transmission of COVID-19. ^2^ Higher wind speeds in external settings contribute to dilution and droplet removal, resulting in a decline in airborne concentration ( https://apps.who.int/iris/bitstream/handle/10665/70863/WHO_CDS_CSR_GAR_2003.11_eng.pdf?sequence=1&isAllowed=y). ^20^ In the internal environment, higher wind speeds decrease the viral load indoors, which can prevent SARS outbreaks. ^21^ Other researchers have found that high wind speeds can actually suppress the spread of COVID-19 cases. ^22^^–^^24^ Therefore, the variable also serves as a potential consideration in suppressing disease transmission. ^22^

This study considers some of the limitations of previous studies by examining the potential of wind speed in influencing the spread of cases based on regional characteristics ^14^^,^^16^^,^^19^^,^^22^^,^^24^ because environmental conditions between regions are different. ^25^ In addition, daily COVID-19 case data were converted into weekly cases to adjust for the incubation period of COVID-19 (5-6 days) and the uncertain time interval between the first day of infection and the day of sampling. ^26^

Based on the description above, it can be assumed that wind speed is a major influencing factor of COVID-19 spread. Therefore, it was crucial to comprehend the effects of wind speed on the virus, as Jakarta appeared to be the pandemic's epicenter. Consequently, this research successfully investigated the relationship between wind speed and the weekly occurrence of COVID-19, as well as the potential spread of cases based on regional characteristics in Jakarta, especially in coastal areas.

## Methods

A step by step description of the protocols can be obtained at: dx.doi.org/10.17504/protocols.io.ewov1odzylr2/v1. ^27^

### Sample and data

The secondary data comprised daily reports of COVID-19 infection and wind speed (maximum and mean) in Jakarta from the pandemic inception, specifically between March and September 2020. These data were obtained from case reports of hospitals, health centers, and laboratories recorded per urban village by the Jakarta Provincial Health Office and can be accessed on the Jakarta Provincial Health Office website ( https://corona.jakarta.go.id/id/data-pemantauan) and reports of two weather monitoring stations (Kemayoran station and Tanjung Priok station) in Jakarta, which can be accessed on the website of the Meteorology, Climatology, and Geophysics Agency ( https://dataonline.bmkg.go.id/akses_data). Subsequently, the general information was converted into 31-weeks documentation. The conversion of daily cases to weekly cases is related to several considerations, such as the incubation period of COVID-19, which lasts for 5–6 days on average, and the uncertain time interval between the first day of infection and the day of sampling. ^26^ Furthermore, a basic map of Jakarta with neighboring community boundaries was obtained using the GADM Map and Data site ( https://gadm.org/maps/IDN/jakartaraya.html). Coordinates for the weather monitoring stations were accessed online ( https://www.gps-latitude-longitude.com/). The Jakarta province comprising 261 urban villages served as the research location.

### Measures of variables

The unit of analysis in this study was all urban villages in Jakarta. A quantitative method was applied with an ecological design that provides real-time and location analysis using geographic information systems, and the data were tested using statistical techniques. The study focuses on the following measures:

• Maximum and average wind speed in m/s in Jakarta from March to September 2020.
• COVID-19 cases. The number of weekly COVID19 cases in Jakarta from March to September 2020.

### Models and data analysis procedure

Univariate analysis was conducted to determine individual variable distribution, including maximum and average wind speed (m/s), as well as the number of COVID-19 cases. This process is descriptive and quantitative, where the data exist in statistical distribution tables, line graphs, and thematic maps based on the research objectives. Before the correlation test is applied, the data normality test is conducted first. The results of the normality analysis show that the maximum wind speed variable is normally distributed, while the average wind speed and COVID-19 cases are not normally distributed. From these results, variables that are not normally distributed are then transformed by the square root (x) to obtain normality. This step is taken to fulfill the requirements for multiple linear regression analysis (normality assumption). Subsequently, the bivariate analysis involved Pearson's product-moment correlation test to evaluate the relationship between independent (wind speed factor) and dependent variables (COVID-19). Specifically, the method stated the possible existence (p < 0.05), closeness (r), and direction of the relationship. In addition, the strengths of the association were qualitatively divided into four categories, where r = 0.00–0.25 was absence/weak relationship, r = 0.26–0.50 was moderate, r = 0.51–0.75 was strong and r = 0.76–1.00 was very strong/perfect. ^28^ This categorization aims to determine how strongly the independent variables (maximum wind speed and average wind speed) correlate with the dependent variable (COVID-19 cases). It is also used to discuss the results of the analysis in the discussion section. The correlation value also determined the direction of the relationship as a positive (+) or negative (−) pattern. This value (r) was evaluated by the conditions, where r = 0 was no linear relationship, r = −1 was perfect negative linear and r = 1 was perfect positive linear. ^28^ Furthermore, the univariate and bivariate analyses were conducted at the Computer Laboratory using SPSS 21 software (RRID:SCR_002865).

Spatial analysis was performed to observe the relationship pattern between the two variables. Based on a selected community, an interpolation process was employed to create an overlay map of COVID-19 cases and climate parameters. The Jakarta grid map interpolation was used to estimate the magnitude of climate variables outside the measurement points (weather stations) by applying the following steps. Firstly, a grid map of five weather monitoring stations was created. The interpolation was performed by entering the point values or coordinate attribute data (longitude and latitude) into the climate variable attribute table. The coordinate points were joined in the climate variable map. Secondly, the independent variable vector data were digitized by inputting the spatial data on climate variables into a base map, then processing and selecting a color symbol (singleband pseudocolor) with color ramp blues. Consequently, a digital category of high and low climate variables was formed depending on the data magnitude. Thirdly, the dependent variable vector data were digitized by entering spatial data on COVID-19 rates into the base map, depending on the community, followed by processing and selecting a point symbol (centroid). A digital category of large and small cases was generated based on the disease data. Fourthly, the two vector maps were interpolated with the plugin interpolation menu. Therefore, an interpolated raster plot was obtained and used to analyze or predict the climate variable values in each community. The resulting color gradations and point symbols did not show any ratio but only reported ordinal values, including high-low climate variations and number of virus cases. This color gradation ranged from dark blue to white, indicating high to low wind speeds (maximum and mean). Subsequently, the colors were created digitally using a singleband pseudocolor with ramp blues colors from Quantum Geographic Information System (QGIS) software (RRID:SCR_018507) with a natural grouping of five classes, where very dark blue = very high, dark blue = high, blue = medium, light blue = and white = very low. The dot symbol (centroid) varied from large to small, representing the virus spread. Similarly, the point symbol size was digitally generated using a simple marker or a standard symbol from the QGIS software with a linear classification between 0 and 17.

The spatially analyzed data were further processed with overlayed thematic graphics and maps to show the relationship pattern based on time and location. The spatial analysis was associated with the statistical correlation results generated using the QGIS software version 3.0 at the Computer Laboratory of the Faculty of Public Health, University of Indonesia.

## Results

The wind speed (maximum and mean) from March to September 2020 tended to fluctuate between 1.43 and 6.07 m/s. This variable appeared minimal during the last week of each month (fourth, fifth, fourth, fourth, and fifth weeks of March, April, May, June, and July, respectively). However, it showed a higher performance in the middle of each month (second, second, second, third, third, and third weeks of March, May, June, July, August, and September, respectively).

Furthermore, the maximum and minimum wind speed values of 6.07 and 4.5m/s occurred during the first, ninth, and 15 ^th^ weeks, respectively. Meanwhile, the optimal and minimum average wind speeds of 2.75 and 1.43 m/s were obtained in the 29 ^th^ and fourth weeks, respectively.

**Figure 1.**
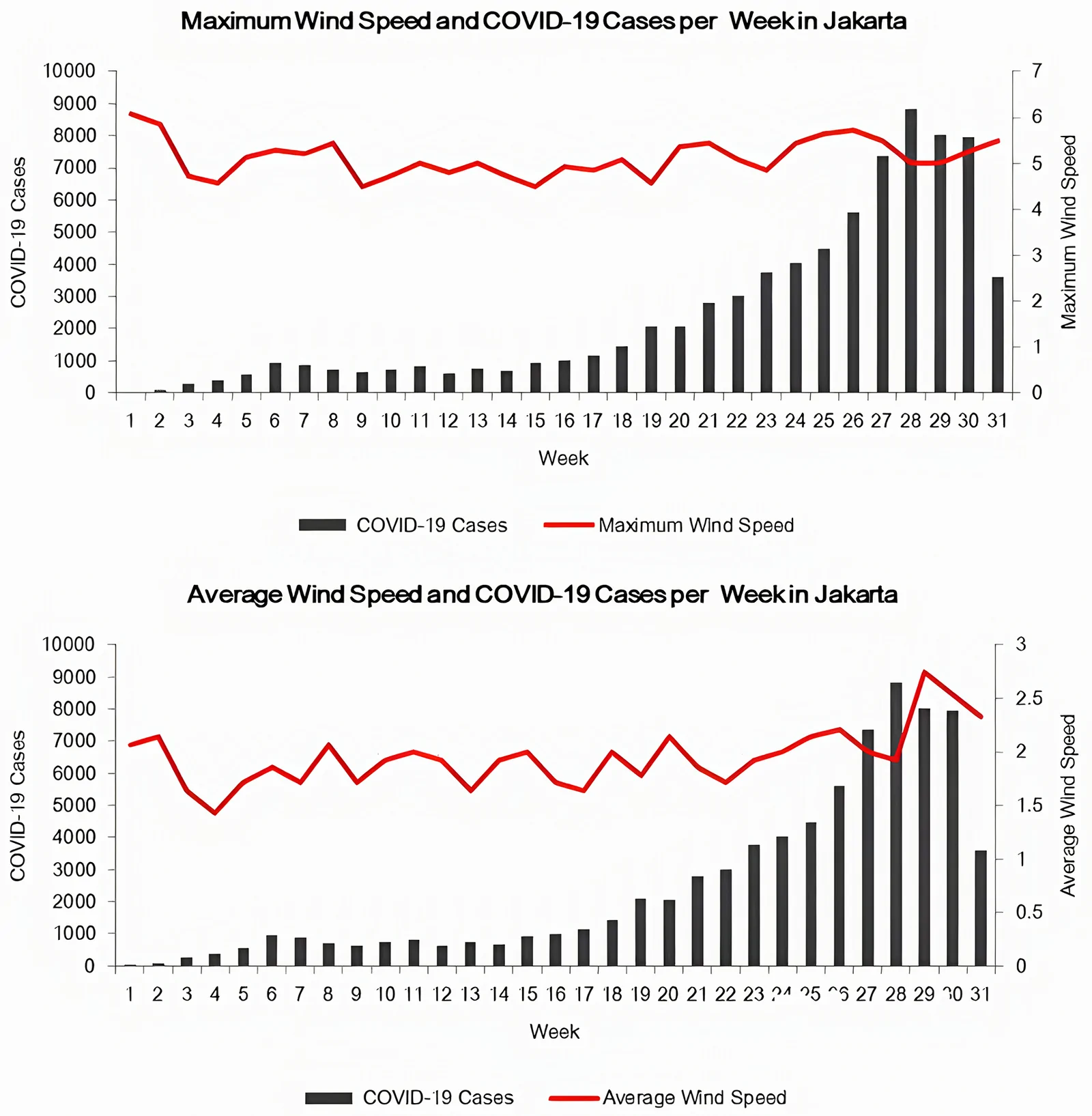
Wind speed (maximum and average) and COVID-19 cases per week in March-September 2020 in Jakarta (accessed from: https://dataonline.bmkg.go.id/akses_data and https://corona.jakarta.go.id/id/data-pemantauan).

The Pearson product-moment correlation test was applied in the statistical analysis of the relationship to show the occurrence (p < 0.05), direction (positive/direct or negative/opposite), and closeness (r). The Jakarta data from March to September 2020 were processed. Table 1 shows that the average wind speed and COVID-19 cases have a strong and positive correlation (p = 0.002, r = 0.542). These conditions indicated that higher average wind speeds increased the possibility of COVID-19 occurrence. However, no significant correlation existed between the maximum wind speed and the virus occurrence. Figure 1 shows that wind speeds possibly generated a pattern similar to the COVID-19 cases. The reverse form of the maximum wind speed with COVID-19 cases occurred in the second to fourth, eighth, 15th, 17th, 19th, 22nd, 23rd, 27th, 28th, 30th, and 31st weeks, while for average wind speed, the pattern was observed at weeks three, four, seven, nine, 13, 14, 16, 17, 19–22 and 27–29.

**Table 1.** Pearson’s correlation coefficient between wind speed and COVID-19.

Wind speed variables	Pearson’s correlation coefficient
Maximum wind speed	0.164
Average wind speed	0.542 *

* Correlation is significant at the 0.01 level (2-tailed).

The spatial analysis was created by overlaying a map of COVID-19 cases with a plot of the sunlight duration to obtain the interpolation from the weather monitoring stations. Color gradations and dot symbols represent this interval and the virus occurrence. In addition, the interpolated values were used to predict a relationship between sunlight duration and COVID-19 in each village around the research location.

Apart from generating a high-low comparison of sun exposure to the number of COVID-19 cases, the virus transmission pattern from March to September 2020 was also ascertained. There were incomplete data, including the records per village, that commenced from March 25, 2020. This circumstance tended to influence the distribution pattern in March, causing a uniform condition to be a low category.

In each month, the spatial pattern of maximum wind speed tended to fluctuate, including the maximum high wind speed dominating the central and northern parts of the capital city in the first two months (March and April). Subsequently, the flow expanded to the northeast and northwest regions, comprising Tanjung Priok, Koja, Kebon Bawang, Lagoa, Kali Baru, North Rawabadak, South Rawabadak, Warakas, Papanggo, North Tugu, West Semper, Cilincing, and East Semper communities. The maximum moderate wind speed consistently occurred in the southeastern part that included most of the urban villages in East Jakarta. In contrast, low maximum wind speeds were reported in the southwest part relating to the urban villages in South Jakarta.

**Figure 2.**
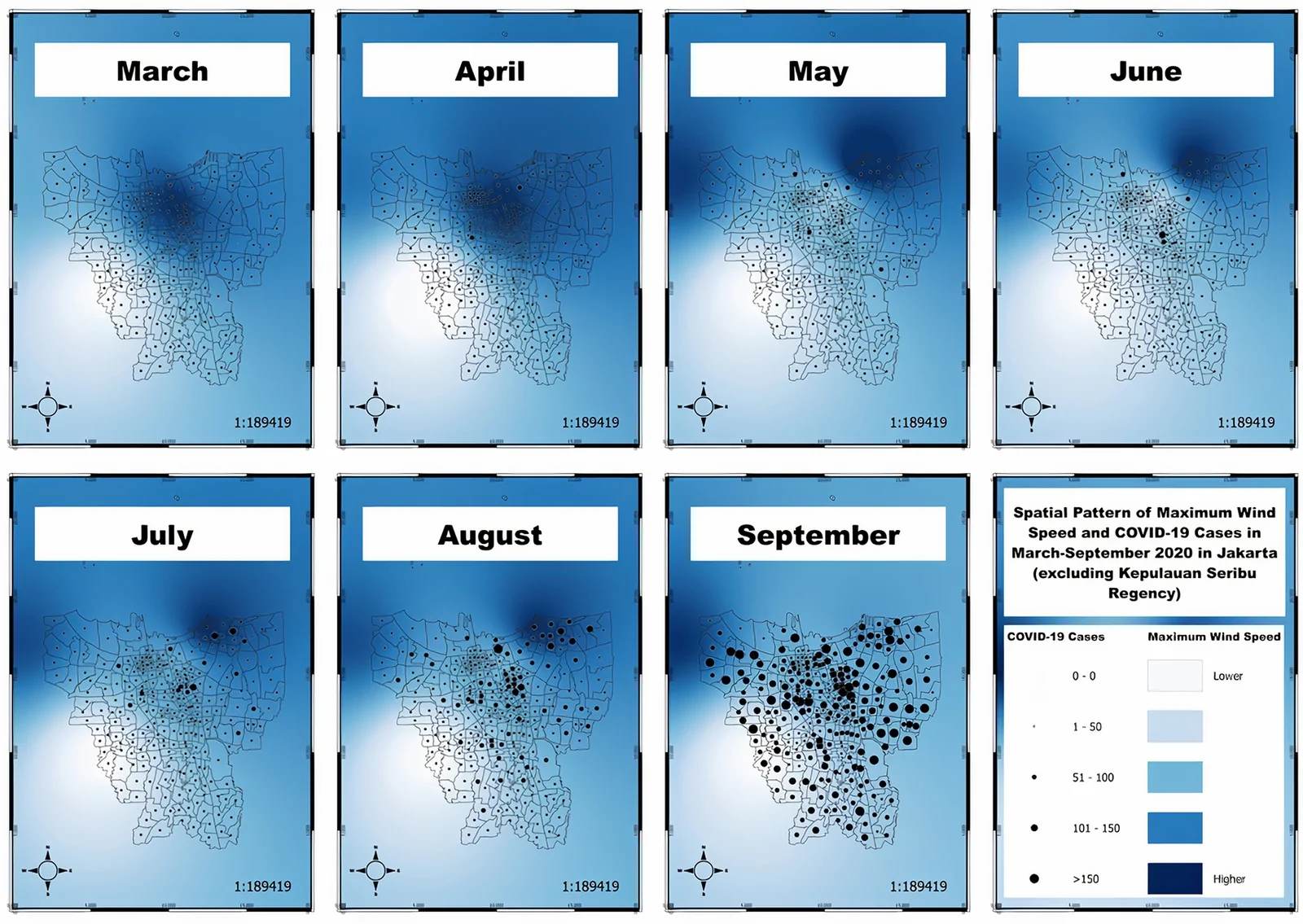
Maximum wind speed spatial pattern with COVID-19 cases from March to September 2020 (accessed from: https://dataonline.bmkg.go.id/akses_data and https://riwayat-file-covid-19-dki-jakarta-jakartagis.hub.arcgis.com/).

Based on the spatial map overlaying the maximum wind speed and COVID-19 cases, urban villages with high maximum wind speeds tended to demonstrate faster virus transmission than areas with moderate and low maximum wind speeds. A relatively high spike in cases in July circulated across the villages of Lagoa (101) and Kebon Bawang (10), and in August at Lagoa (144), Cilincing (131), South Rawabadak (105), and West Semper (109), compared with other neighboring villages. However, a small number of villages with moderate and low maximum wind speeds also showed an increase in July at West Cempaka Putih (101), and in August at Johar Baru village (127), and West Cempaka Putih (106), as illustrated in Figure 2.

The spatial pattern of the average wind speed in each month appeared relatively similar, including the high average wind speed dominating the northeast and northwest regions of the city. This area covered Kebon Bawang, Tanjung Priok, Koja, Lagoa, Kali Baru, North Rawabadak, South Rawabadak, Warakas, Papanggo, North Tugu, West Semper, Cilincing, Kramat, Tegal Alur, Pengadungan, Kali Deres, and Samanan. Furthermore, the average wind speed consistently occurred in the areas with high and low average wind speed convergence, including Kapuk Muara, Kapuk, Rawa Buaya, Duri Kosambi, Sunter Agung, Sunter Jaya, West Kelapa Gading, East Kelapa Gading, and Pegangsaan Dua. The low average wind speeds also remained constant in the central and southern parts, including central, south, and east Jakarta.

**Figure 3.**
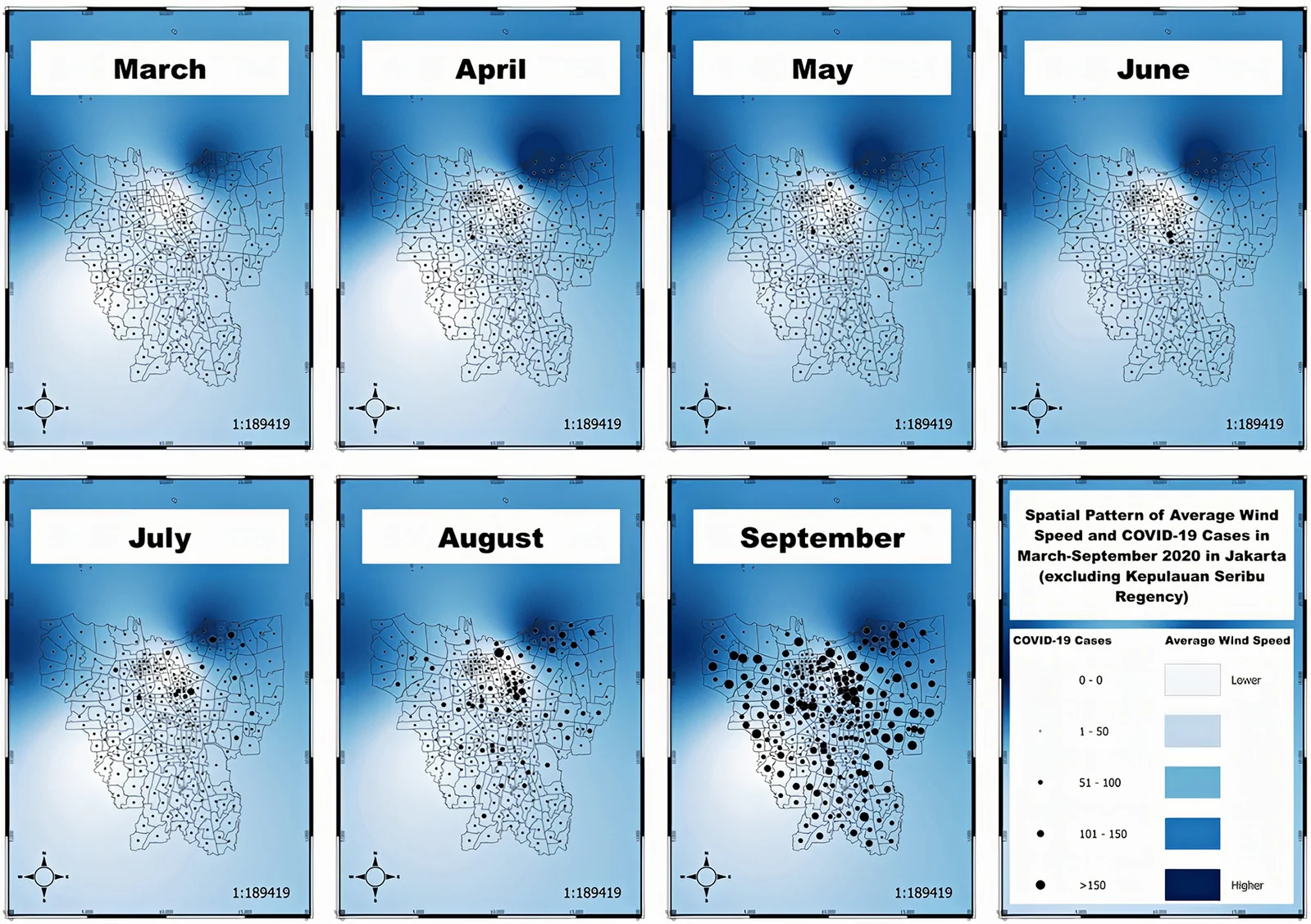
Spatial pattern of average wind speed with COVID-19 cases from March to September 2020 (accessed from: https://dataonline.bmkg.go.id/akses_data and https://riwayat-file-covid-19-dki-jakarta-jakartagis.hub.arcgis.com/).

Based on the spatial overlay map of the average wind speed and COVID-19 cases, villages with high average wind speeds experienced an increase of cases faster compared with areas with medium and low average wind speeds. A relatively high spike also occurred in July at Kebon Bawang (101) and Lagoa (101), and in August at Lagoa (144), Cilincing (131), West Semper (109), and South Rawabadak (105), compared with other adjacent villages. However, a smaller number of villages with medium and low average wind speeds also showed an increase in July at West Cempaka Putih (101), and in August at West Pademangan (158), Johar Baru (127), and West Cempaka Putih (106), as represented in Figure 3.

## Discussion

In this study, we found that average wind speed correlated with COVID-19 cases in Jakarta by accelerating the transmission of the virus. This outcome matched several previous studies, ^14^^–^^19^^,^^22^^–^^24^ where the wind speed was also known to increase the airborne SARS-CoV-2 spread. ^14^ Droplets released during normal speech tend to survive in the air between eight and 14 minutes, similar to confined environments. ^1^ Recent studies have also shown that the virus can remain infectious in airborne particles beyond three hours. ^10^^,^^11^Therefore, aerosol transmission appears to be the main channel, compared with other media. ^1^^,^^3^^,^^4^ Under these circumstances, the wind speed variable plays a significant role in promoting the spread, ^19^ and with wind speeds in Jakarta ranging from 1.43 to 6.07 m/s, the surviving airborne virus quickly spreads. ^14^

In this context, higher wind speeds adversely impact individuals who do not correctly apply health protocols (avoiding crowds, not keeping close distances, and not wearing/removing masks). ^19^ Therefore, the transmission of impure cases is influenced by the rate of wind speed spreading the virus. Furthermore, government policies also play an essential role in influencing the disease rates, in the form of implementing health protocols (washing hands, ^29^ wearing masks, ^29^ physical distancing ^30^), staying at home, ^31^ working from home, ^32^^,^^33^ and large-scale social restrictions/enforcement of restrictions on community activities (Pembatasan Sosial Berskala Besar/Pemberlakuan Pembatasan Kegiatan Masyarakat). ^34^^–^^36^

Based on a weekly graphical analysis of Jakarta, the fluctuations in wind speed tended towards the pattern of COVID-19 cases. The variable results were also consistent with similar outcomes of the correlation test in Jakarta, indicating a significant relationship with strong and positive patterns. This also indicated that higher average wind speeds triggered extensive COVID-19 spread.

The spatial relationship between wind speed and COVID-19 cases in Jakarta from March to September 2020 showed that urban villages that had high wind speeds tended to experience a faster transmission than other areas with medium and low values. However, a smaller number of villages with moderate and low wind speeds also showed increased cases. This result was possibly influenced by determining the source location of the COVID-19 cases using the domicile/residential address in the last 14 days, despite the possible occurrence of the infection while conducting external activities. Therefore, the spatial pattern of COVID-19 cases in Jakarta did not match the actual spatial pattern from the infection origin. ^26^ In addition, other factors, including community non-compliance in implementing health protocols and *Pembatasan Sosial Berskala Besar* (PSBB) or large-scale social restrictions policies, led to transmissions outside the home ( https://www.liputan6.com/news/read/4368373/survei-bps-55-persen-masyarakattak-patuhi-protokol-kesehatan-karena-tidak-ada-sanksi). The previous survey proved that approximately 26.46% of the community did not correctly implement health protocols outside the home ( https://www.bps.go.id/publication/2020/09/28/f376dc33cfcdeec4a514f09c/perilaku-masyarakat-di-masa-pandemi-covid-19.html). Therefore, strict health protocols (washing hands, wearing masks, physical distancing), implementation of maximum capacity, and operating hours rules in places with open-air conditions and crowds, particularly tourist attractions in areas with high wind speeds, including coastal areas, are among several considerations for policymakers.

The majority of the previous studies employed case and wind speed data on similar days, although the reporting record did not represent the infection date, as the symptoms typically manifested after days of transmission. The time required for tracking and testing was equally essential, causing difficulty in determining the actual infection date. Therefore, to minimize possible bias, daily case and wind speed data were converted to a weekly form, based on a mean incubation period (symptom appearance) of between five and six days, with a maximum of 14 days. ^26^

This study is expected to consider certain limitations that may have influenced the overall results. The limited period of data collection (seven months) possibly influenced the analysis. No comparative analysis of the relationship between wind speed variables and case variables was performed weekly, every 14 days, or monthly, indicating that the time frames were closely related. Neither were the analyses conducted using time lags, and therefore, it was not possible to determine the most significant relationship between wind speed and COVID-19 case variables between week one (t) and subsequent intervals ((t+1), (t+2), and so on). In addition, the unavailability of variable data in smaller regions, neighborhood/community associations ( *Rukun Tetangga*/ *Rukun Warga*), produced indefinite distribution patterns.

In terms of other perspectives, this study also has limitations as it did not include other specific factors, which also influence the accelerated increase in infections, such as air pollution levels, economic wealth, cultural characteristics, ^2^ population size, density, mobility and immunity, community behavior (washing hands, wearing masks, and physical distancing), and the nature of the virus were not included in this research. ^2^^,^^29^^,^^30^ Low wind speeds are associated with a high concentration of air pollutants, and therefore may promote a longer permanence of viral particles in polluted air of cities, thus favoring an indirect means of diffusion of the novel coronavirus (SARS-CoV-2). ^37^ Proactive strategies to address similar pandemics should also be designed from an environmental and socio-economic perspective, which is also based on sustainability science and environmental science. ^2^ Therefore, further studies are expected to consider the above mentioned factors, particularly in areas with similar characteristics to achieve comprehensive results.

## Conclusions

Based on the overall results, areas with high wind speeds tended to show an increase in the number of COVID-19 cases, especially in the coastal areas of Jakarta. Wind speed plays a role in increasing the spread of SARS-CoV-2, in people who did not implement health protocols properly. Therefore, the impure cases are triggered by the rate of the wind-speed-based transmission. Consequently, strict health protocols (washing hands, wearing masks, physical distancing), applying maximum capacity, and regulating work hours in places with open-air conditions and crowd potentials, especially tourist attractions in areas with high wind speeds, including coastal regions, serve as a basis for consideration in policymaking.

The limitation of the study that did not include other influential factors, such as air pollution levels, economic wealth, cultural characteristics, etc., is important to be included in future research to provide policy makers with comprehensive recommendations from the perspective of environmental, socio-economic, and other factors, in order to create programs that can prevent similar pandemics in the future.

## Ethical approval

This research was approved by the Research and Community Engagement Ethical Committee, Faculty of Public Health, Universitas Indonesia, No. 210/UN2.F10. D11/PPM.00.02/2021.

## Data availability

### Underlying data

Dryad. Wind Speed Data. https://datadryad.org/stash/dataset/doi:10.5061/dryad.41ns1rnj9. ^38^

This project contains the following underlying data:

• Wind_speed_data1.csv
• README_wind_sd.md

Special Capital Region of Jakarta Provincial Health Office COVID-19. https://corona.jakarta.go.id/id/data-pemantauan. ^39^

• Data on Covid-19 cases

Meteorology, Climatology, and Geophysics Agency. https://dataonline.bmkg.go.id/akses_data. ^40^

• Wind speed data

GADM Map and Data. https://gadm.org/maps/IDN/jakartaraya.html. ^41^

• The base map for the Special Capital Region of Jakarta

Longitude and Latitude/GPS coordinates. https://www.gps-latitude-longitude.com/. ^42^

• Coordinates of the weather monitoring station

The spatial analysis of maximum and average wind speed and COVID-19 March-September 2020. https://figshare.com/s/8578f3944f4fe3908e6c ^43^
