# Supplementary material for: The effect of wind speed in increasing COVID-19 cases in Jakarta: a spatial-temporal analysis from March to September 2020
Source: F1000Res. 2023 Dec 28;12:145. Originally published 2023 Feb 8. [Version 3] doi: 10.12688/f1000research.128908.3 (PMC13560010; doi:10.12688/f1000research.128908.3)
Supplement: Peer review history

# Reviewer report: Reviewer response for version 3

**DOI:** 10.5256/f1000research.160351.r509845

**Published:** 2026-09-08

**Authors:** Jumadi Jumadi

**Affiliations:** 1 Universitas Muhammadiyah Surakarta, Surakarta, Central Java, Indonesia

**Review Report**

**Manuscript:** *The Effect of Wind Speed in Increasing COVID-19 Cases in Jakarta: A Spatial-Temporal Analysis from March to September 2020*

 In general, while the manuscript is technically sound, its current contribution is limited by the declining timeliness of the topic and a relatively simple analytical framework. Substantial methodological strengthening, particularly through appropriate spatial and spatial-temporal statistical analysis, is necessary to establish a more convincing geographical contribution. However, several substantive concerns should be addressed to strengthen its relevance and geographical contribution.

1. **Timeliness and scientific contribution.** The study focuses exclusively on COVID-19 transmission during March–September 2020. Given the substantial body of literature that has subsequently emerged on COVID-19, meteorological factors, and spatial epidemiology, the topic is now **less timely**. The authors therefore need to clearly explain why revisiting this early pandemic period remains scientifically important. The Introduction and Discussion should engage with more recent literature and demonstrate what new knowledge this study provides beyond the numerous studies already examining weather–COVID-19 relationships.
2. **Statistical analysis is too simple.** The current analytical framework is insufficient to support a strong spatial-temporal contribution. Simple statistical associations between wind speed and COVID-19 cases do not adequately account for the spatial dependence, spatial heterogeneity, and temporal dynamics inherent in the data. The analytical framework therefore requires substantial improvement.
3. **Strengthen spatial analysis and spatial statistics.** Since the manuscript is presented as a spatial-temporal study, the authors should incorporate appropriate **spatial statistical methods**. Depending on the data structure and research objectives, this may include global and local spatial autocorrelation (e.g., Moran’s I/LISA), hotspot analysis, spatial regression, geographically weighted approaches, or spatial-temporal models. Potential confounding factors such as population density, mobility, urban morphology, and other meteorological variables should also be considered where data are available. Importantly, the selected methods should be theoretically justified rather than added merely to increase methodological complexity.

Is the work clearly and accurately presented and does it cite the current literature?

Partly

If applicable, is the statistical analysis and its interpretation appropriate?

Yes

Are all the source data underlying the results available to ensure full reproducibility?

Partly

Is the study design appropriate and is the work technically sound?

Yes

Are the conclusions drawn adequately supported by the results?

Yes

Are sufficient details of methods and analysis provided to allow replication by others?

Partly

Reviewer Expertise:

GIS

I confirm that I have read this submission and believe that I have an appropriate level of expertise to confirm that it is of an acceptable scientific standard, however I have significant reservations, as outlined above.

# Reviewer report: Reviewer response for version 3

**DOI:** 10.5256/f1000research.160351.r509844

**Published:** 2026-08-20

**Authors:** Pandji Wibawa Dhewantara

**Affiliations:** 1 National Research and Innovation Agency of The Republic Indonesia, Cibinong, Indonesia

The study aimed to examine the role of wind speed in COVID-19 dynamics in Jakarta, Indonesia, using an ecological spatial-temporal approaches. The study demonstrates that wind-speed was correlated with COVID-19 patterns in Jakarta during March to September 2020. Overall, the paper is well written and highlights an interesting finding. However, there are several points that should be addressed to improve the clarity and quality of the manuscript before indexation.

 Specific comments:

 - Introduction: Paragraph (par) 1 - please update the wording, COVID-19 case data and specific time. Using narrative "are currently experiencing a significant increase" seems now not relevant for Indonesia. Better to remove or rephrase this sentence. 

 - Introduction Par. 5 - There's study in Jakarta also found that low wind speed associated with an increase COVID-19 case during March-May 2020 (see Rendana M. Impact of the wind conditions on COVID-19 pandemic: A new insight for direction of the spread of the virus. Urban Clim. 2020 Dec; 34:100680. doi: 10.1016/j.uclim.2020.100680). So better to add this in the text and discussion.

 - Introduction Par. 7 --- "...comprehend the effects of wind speed on the virus...." Better to rephrase "effects of wind speed to the spread of the COVID-19 cases or the distribution of COVID-19 cases". Please use this term throughout the manuscript.

 - Methods: Please add a description of the Study Site (add a subsection "Study site")

 - Methods: The authors used COVID-19 data from March to September 2020. If possible, it's better to extend the period of observation (e.g up to one year or more) so we can capture whether there was an interaction between season, wind speed, and COVID-19 trend. Adding more observations will refine the findings. Please consider this.

 - Methods: Please add sub-headings for each analysis (e.g., Descriptive analysis, Correlation analysis, and Spatial analysis) to improve readability.

 - Results:  Figure 2 and Figure 3 - Just to clarify, was COVID-19 case as illustrated on both figures represent total "new" cases for each month for every village OR a cumulative case for COVID-19 from March to September?

 - Discussion: Par 1., first line: should add the period of observation. Suggestion: In this study, we found that average wind speed was correlated with COVID-19 spread in Jakarta during March to September 2020.

 - Discussion: Par. 3, first line: ...the fluctuations in wind speed tended towards the pattern of COVID-19. What does it mean? Not clear. Please rephrase.

 - Discussion: Par 6. Limitations -- the author stated that the limited period of data may influence the analysis (as per 2020/2021 when the study was conducted). As we are now in 2026 -- it is now possible to add or analyze more data/observation to better look whether wind speed affect the spatial - temporal patterns of COVID-19 in Jakarta. 

 - Conclusions - Par 2 -- it has been stated in the Discussion (last Par). Please remove.

Is the work clearly and accurately presented and does it cite the current literature?

Partly

If applicable, is the statistical analysis and its interpretation appropriate?

Yes

Are all the source data underlying the results available to ensure full reproducibility?

Yes

Is the study design appropriate and is the work technically sound?

Yes

Are the conclusions drawn adequately supported by the results?

Yes

Are sufficient details of methods and analysis provided to allow replication by others?

Partly

Reviewer Expertise:

infectious diseases epidemiology, spatial statistics, climate and health, one health

I confirm that I have read this submission and believe that I have an appropriate level of expertise to confirm that it is of an acceptable scientific standard, however I have significant reservations, as outlined above.

## References

[1] : Impact of the wind conditions on COVID-19 pandemic: A new insight for direction of the spread of the virus. *Urban Climate* .2020;34: 10.1016/j.uclim.2020.100680 10.1016/j.uclim.2020.100680 PMC740227932834966

[2] : Correlation between weather and Covid-19 pandemic in Jakarta, Indonesia. *Science of The Total Environment* .2020;725: 10.1016/j.scitotenv.2020.138436 10.1016/j.scitotenv.2020.138436 PMC727084732298883

[3] : Investigating the association between PM2.5, Climate variables, and COVID-19 daily reported cases from March 2020 to November 2021 in El Paso County, Texas: A Time-Series Analysis. *Environmental Science and Pollution Research* .2026;33(8) : 10.1007/s11356-026-37413-83206-3219 10.1007/s11356-026-37413-8 41652267

[4] : COVID-19 and its association with meteorological, climate, and environmental factors: An umbrella review. *Journal of Public Health Research* .2025;14(3) : 10.1177/22799036251358298 10.1177/22799036251358298 PMC1230458240735053

# Reviewer report: Reviewer response for version 3

**DOI:** 10.5256/f1000research.160351.r233973

**Published:** 2024-01-23

**Authors:** Mario COCCIA

**Affiliations:** 1 CNR -- National Research Council of Italy, Collegio Carlo Alberto, Moncalieri, Italy; 2 Arizona State University (Ringgold ID: 7864), Tempe, arizona, USA

I have read thoroughly the revised version of paper.

 The authors have done considerable additional work, and addressed all concerns and criticisms in the revised manuscript, which I believe has improved substantially in the theoretical framework, study design and discussion of results.

Is the work clearly and accurately presented and does it cite the current literature?

Partly

If applicable, is the statistical analysis and its interpretation appropriate?

Partly

Are all the source data underlying the results available to ensure full reproducibility?

Partly

Is the study design appropriate and is the work technically sound?

Partly

Are the conclusions drawn adequately supported by the results?

Partly

Are sufficient details of methods and analysis provided to allow replication by others?

Partly

Reviewer Expertise:

COVID-19 and Environment

I confirm that I have read this submission and believe that I have an appropriate level of expertise to confirm that it is of an acceptable scientific standard.

# Response

**Published:** 2025-10-25

**Authors:** Dewi Susanna

**Affiliations:** Universitas Indonesia, Indonesia

Dear Mario COCCIA,

 Thank you for accepted our responses.

 With warm regards,

 Dewi

# Reviewer report: Reviewer response for version 2

**DOI:** 10.5256/f1000research.153848.r203833

**Published:** 2023-11-02

**Authors:** Mario COCCIA

**Affiliations:** 1 CNR -- National Research Council of Italy, Collegio Carlo Alberto, Moncalieri, Italy; 2 Arizona State University (Ringgold ID: 7864), Tempe, arizona, USA

Analysis of the effect of wind speed in increasing the COVID-19 cases in Jakarta

 The topics of this paper are interesting, though well known. The structure and content must be revised, and results have to be better explained by authors before to be reconsidered for indexing.

 Title has to clarify the period under study. 

 Abstract has to clarify the goal and environmental, health and social implications. 

 Introduction has to better clarify the research questions of this study and provide more theoretical background. Authors have to better describe the different sources of transmission dynamics of COVID-19 (e.g., climate, air pollution, density, etc.) and risk factors in society, which can accelerate diffusion of this novel coronavirus in environment. The study of wind speed has to be associated with temperature and air pollution to well explain transmission of COVID-19 and similar diseases. After that they can focus on the topics of this study to provide a correct analysis for fruitful discussion (See suggested readings that must be all read and used in the text). 

 Methods of this study is not clear. The section of Materials and methods must be re-structured with following three sections only and same order:

- Sample and data
- Measures of variables
- Models and Data analysis procedure. 

Results

- Authors have to avoid a lot of subheadings that create fragmentation and confusion. If necessary, can use bullet points (same comments for section of results and all sections). 
- Figure 1 has to indicate the year in title to be clear. 
- The two graphs can be merged in a single figure with two lines to be clear. Figures 2 and 3 are so small that cannot be read. Provide a note to the figure with more description. Or put them in appendix with larger size per each month. 

Discussion. 

 First, authors have to synthesize the main results in a simple table to be clear for readers and then show what this study adds compared to other studies. 

 Conclusion so short it is useless. Conclusion has not to be a summary, but authors have to focus on manifold limitations of this study and provide suggestions of environmental, health, crisis management and social policy, as well as how nations can prevent with good environmental and health policies. 

 Overall, then, the paper is interesting, but structure is confused. Theoretical framework is weak, and some results create confusion… structure of the paper has to be improved; study design, discussion and presentation of results have to be clarified using suggested comments.

 I strongly suggest improving the paper, by using all comments (suggested papers included to read and use all ^1^^-^^18^) and maybe it can be considered.

 In particular, an accurate reading and use of following studies can reinforce the theoretical framework of this study, support the discussion of research in a comparative analysis of other context studied, so authors can provide fruitful implications for policymaker to design an health policy to face pandemic crisis in context as Jakarta and cities having similar climate, environmental and atmospheric factors.

 Suggested readings of relevant papers that have to be read and all inserted in the text and references ^1^^-^^18^.

Is the work clearly and accurately presented and does it cite the current literature?

Partly

If applicable, is the statistical analysis and its interpretation appropriate?

Partly

Are all the source data underlying the results available to ensure full reproducibility?

Partly

Is the study design appropriate and is the work technically sound?

Partly

Are the conclusions drawn adequately supported by the results?

Partly

Are sufficient details of methods and analysis provided to allow replication by others?

Partly

Reviewer Expertise:

COVID-19 and Environment

I confirm that I have read this submission and believe that I have an appropriate level of expertise to confirm that it is of an acceptable scientific standard, however I have significant reservations, as outlined above.

## References

[1] : COVID-19 and air pollution and meteorology-an intricate relationship: A review. *Chemosphere* .2021;263: 10.1016/j.chemosphere.2020.128297128297 33297239 10.1016/j.chemosphere.2020.128297 PMC7487522

[2] : Sources, diffusion and prediction in COVID-19 pandemic: lessons learned to face next health emergency. *AIMS Public Health* .2023;10(1) : 10.3934/publichealth.2023012145-168 37063362 10.3934/publichealth.2023012 PMC10091135

[3] : Association between ambient temperature and COVID-19 infection in 122 cities from China. *Sci Total Environ* .2020;724: 10.1016/j.scitotenv.2020.138201138201 32408450 10.1016/j.scitotenv.2020.138201 PMC7142675

[4] : Preparedness of countries to face COVID-19 pandemic crisis: Strategic positioning and factors supporting effective strategies of prevention of pandemic threats. *Environ Res* .2022;203: 10.1016/j.envres.2021.111678111678 34280421 10.1016/j.envres.2021.111678 PMC8284056

[5] : Correlation between environmental pollution indicators and COVID-19 pandemic: A brief study in Californian context. *Environ Res* .2020;187: 10.1016/j.envres.2020.109652109652 32405084 10.1016/j.envres.2020.109652 PMC7219392

[6] : SARS-CoV-2 and other pathogenic microorganisms in the environment. *Environmental Research* .2021;201: 10.1016/j.envres.2021.111606 10.1016/j.envres.2021.111606 PMC845933434181924

[7] : Impact of the COVID-19 Event on Air Quality in Central China. *Aerosol and Air Quality Research* .2020;20(5) : 10.4209/aaqr.2020.04.0150915-929 10.4209/aaqr.2020.04.0150

[8] : Global analysis of timely COVID-19 vaccinations: improving governance to reinforce response policies for pandemic crises. *International Journal of Health Governance* .2022;27(3) : 10.1108/IJHG-07-2021-0072240-253 10.1108/IJHG-07-2021-0072

[9] : The impact of first and second wave of the COVID-19 pandemic in society: comparative analysis to support control measures to cope with negative effects of future infectious diseases. *Environ Res* .2021;197: 10.1016/j.envres.2021.111099111099 33819476 10.1016/j.envres.2021.111099 PMC8017951

[10] : Relationship between COVID-19 and weather: Case study in a tropical country. *Int J Hyg Environ Health* .2020;229: 10.1016/j.ijheh.2020.113587113587 32917371 10.1016/j.ijheh.2020.113587 PMC7303605

[11] : Factors determining the diffusion of COVID-19 and suggested strategy to prevent future accelerated viral infectivity similar to COVID. *Sci Total Environ* .2020;729: 10.1016/j.scitotenv.2020.138474138474 32498152 10.1016/j.scitotenv.2020.138474 PMC7169901

[12] : Importance of meteorology in air pollution events during the city lockdown for COVID-19 in Hubei Province, Central China. *Sci Total Environ* .2021;754: 10.1016/j.scitotenv.2020.142227142227 32920418 10.1016/j.scitotenv.2020.142227 PMC7473012

[13] : Can commercial trade represent the main indicator of the COVID-19 diffusion due to human-to-human interactions? A comparative analysis between Italy, France, and Spain. *Environ Res* .2021;201: 10.1016/j.envres.2021.111529111529 34147467 10.1016/j.envres.2021.111529 PMC8459318

[14] : Association between temperature, humidity, and COVID-19 outbreaks in Bangladesh. *Environ Sci Policy* .2020;114: 10.1016/j.envsci.2020.08.012253-255 32863760 10.1016/j.envsci.2020.08.012 PMC7447231

[15] : The effects of atmospheric stability with low wind speed and of air pollution on the accelerated transmission dynamics of COVID-19. *International Journal of Environmental Studies* .2021;78(1) : 10.1080/00207233.2020.18029371-27 10.1080/00207233.2020.1802937

[16] : Impact of meteorological factors on COVID-19 pandemic: Evidence from top 20 countries with confirmed cases. *Environ Res* .2020;191: 10.1016/j.envres.2020.110101110101 32835681 10.1016/j.envres.2020.110101 PMC7442571

[17] : Bidirectional association between COVID-19 and the environment: A systematic review. *Environ Res* .2021;194: 10.1016/j.envres.2020.110692110692 33385384 10.1016/j.envres.2020.110692 PMC7833965

[18] : Pandemic Prevention: Lessons from COVID-19. *Encyclopedia* .2021;1(2) : 10.3390/encyclopedia1020036433-444 10.3390/encyclopedia1020036

# Response

**Published:** 2023-12-18

**Authors:** Dewi Susanna

**Affiliations:** Universitas Indonesia, Indonesia

**Dear Mario Coccia,**

 CNR - National Research Council of Italy, Collegio Carlo Alberto, Moncalieri, Italy

 Thank you very much for your deep review of our manuscript.

 Here are our responses in accordance with your comments as mentioned below:

1. The title has to clarify the period under study. 

*Responses:*

 The title of the article has been revised to " The effect of wind speed in increasing COVID-19 cases in Jakarta: a spatial-temporal analysis from March to September 2020"

1. Abstract has to clarify the goal and environmental, health and social implications.

*Responses:*

 The explanation of objectives in the abstract has been adjusted. Implications for the environment, health and social have been adjusted in the conclusion section.

1. Introduction has to be better clarify the research questions of this study and provide more theoretical background. Authors have to better describe the different sources of transmission dynamics of COVID-19 (e.g., climate, air pollution, density, etc.) and risk factors in society, which can accelerate diffusion of this novel coronavirus in environment. The study of wind speed has to be associated with temperature and air pollution to well explain transmission of COVID-19 and similar diseases. After that they can focus on the topics of this study to provide a correct analysis for fruitful discussion (See suggested readings that must be all read and used in the text). 

*Responses:*

 The introduction has been adjusted with the addition of other sources of COVID-19 transmission dynamics, according to the sources recommended by the reviewer:

 Overall, the transmission dynamics of viral infections, such as COVID-19, are caused by systemic factors (biological characteristics of the virus, incubation period, etc.), specific factors (complex interactions between air pollution, meteorological conditions, etc.), biological characteristics of viral infectivity, and individual health levels (habits, immune system, age, gender, etc.) (Coccia, 2020). The role of climatic elements such as temperature, humidity, precipitation, wind speed, and sunlight are crucial in disease transmission because they are parameters that can determine the survival of the SARS virus in the environment (Rahimi et al., 2021; Rosario et al., 2020; Saputra et al., 2021; Saputra & Susanna, 2021; Srivastava, 2021; Xie & Zhu, 2020).

  

1. Methods of this study is not clear. The section of Materials and methods must be re-structured with following three sections only and same order:
  - Sample and data
  - Measures of variables
  - Models and Data analysis procedure.

*Response:*

 The research method has been adapted to the format recommended by the reviewer.

  

1. Results:
  1. Authors have to avoid a lot of subheadings that create fragmentation and confusion. If necessary, can use bullet points (same comments for section of results and all sections).

*Response:*

 They have been revised in accordance with the recommendations of the reviewers.

  

1.
  1. Figure 1 has to indicate the year in title to be clear.

*Response:*

 The figure-1 is already added.

  

1.
  1. The two graphs can be merged in a single figure with two lines to be clear. Figures 2 and 3 are so small that cannot be read. Provide a note to the figure with more description. Or put them in appendix with larger size per each month.

*Responses:*

- The figures have been revised according to the reviewer's recommendation.
- An appendix of spatial maps per month for each wind speed variable has been created.

 

1. Discussion.  First, authors have to synthesize the main results in a simple table to be clear for readers and then show what this study adds compared to other studies. 

*Responses:*

- It has been adjusted with the recommendations of the reviewers.
- A table of main results has been added.

 

1. Conclusion so short it is useless. Conclusion has not to be a summary, but authors have to focus on manifold limitations of this study and provide suggestions of environmental, health, crisis management and social policy, as well as how nations can prevent with good environmental and health policies.

*Responses:*

 The conclusions have been adjusted with recommendations from the reviewer:

 Based on the overall results, areas with high wind speeds tended to show an increase in the number of COVID-19 cases, especially in the coastal areas of Jakarta. Wind speed plays a role in increasing the spread of SARS-CoV-2, in people who did not implement health protocols properly. Therefore, the impure cases are triggered by the rate of the wind-speed-based transmission. Consequently, strict health protocols (washing hands, wearing masks, physical distancing), applying maximum capacity, and regulating work hours in places with open-air conditions and crowd potentials, especially tourist attractions in areas with high wind speeds, including coastal regions, serve as a basis for consideration in policymaking.

 The limitation of the study that did not include other influential factors, such as air pollution levels, economic wealth, cultural characteristics, etc., is important to be included in future research to provide policy makers with comprehensive recommendations from the perspective of environmental, socio-economic, and other factors, in order to create programs that can prevent similar pandemics in the future.

 Thank you for all the input so this article can be even better.

 With warm regards,

 Authors

# Reviewer report: Reviewer response for version 1

**DOI:** 10.5256/f1000research.141547.r164423

**Published:** 2023-03-24

**Authors:** Edmund I. Yamba

**Affiliations:** 1 Department of Meteorology and Climate Science, Kwame Nkrumah University of Science and Technology (KNUST), Kumasi, Ghana

In this study, the authors examined the effect of wind speed on COVID-19 outcome in Jakarta, Indonesia. Using maximum and mean wind speed data from the Indonesian Meteorology, Climatology and Geophysics Agency; and weekly COVID-19 cases from the special coronavirus website of Jakarta Provincial Health Office, they established the link between wind speed and COVID-19 cases via correlation, graphic/time trend, and spatial analysis. Their results showed that high wind speeds were associated with an increased number of COVID-19 cases, particularly in coastal regions. Hence, the authors concluded that wind speed plays a role in the transmission of COVID-19, especially the coastal areas of Jakarta.

 This study is significant and offers valuable insights into the relationship between wind speed and the transmission of the COVID-19 virus in Jakarta. The findings have practical implications for the implementation of health protocols in areas with high wind speed to mitigate the spread of the virus. The presentation of the study is clear, engaging and comprehensible. Nonetheless, the manuscript requires revision to enhance its quality. Below are my comments regarding areas that need improvement.

**1. The title vs the content presented:** Based on my interpretation, it appears that the authors seek to establish the association between wind speed and COVID-19 cases in Jakarta, without delving into causality, as evidenced by their utilization of Pearson's correlation analysis. The use of the term "effect" in the title implies a notion of causality. Nevertheless, the research work put forth does not explicitly establish causality, but rather investigates the existence of a relationship through inferences. It is recommended that the authors reconsider the phrasing of the title to reflect the content of their study. For example, something like “The relationship between wind speed and COVID-19 infections in Jakarta”.

**2. Introduction/novelty: **Despite the work's overall satisfactory and engaging presentation, the authors have not explicitly addressed the existing research gaps and their contributions to filling them. Previous studies have established the relationship between wind speed and the spread of COVID-19, which raises questions about the motivation behind the authors' replication of this work in Jakarta. Specifically, what were the compelling research questions that drove this inquiry and how were they answered? Hence, the originality or novelty of the work appears to be limited, and the authors should strive to enhance it. One suggestion for improvement could be to extend beyond the typical approach of establishing correlations between weather variables and COVID-19, which is prevalent in most existing literature. Merely identifying a correlation between two variables is insufficient to determine the causative relationship between them. Therefore, panel estimation techniques, for example, could be explored by the authors to investigate the causal aspect further.

**3. Data collection: **The authors conducted a study that involved the collection of daily wind speed and COVID-19 case data spanning March to September 2020, which was then converted into weekly data for 31 weeks. The rationale for the conversion remains unclear, and it would be beneficial if the authors could elucidate their reasons for doing so and how this approach facilitated more effective analysis. Additionally, it is essential to understand how gaps in the data were managed as the quality of the data used is fundamental to the reliability of the results obtained. The authors reported that the study was conducted in Jakarta province, consisting of 261 urban villages. However, it is not evident whether the data used were obtained from each of these villages and aggregated for the province. If this was the case, it would be useful to know whether data were available for each village. It is recommended that the authors provide more comprehensive details regarding the availability and quality of the data used in their analysis to enhance the reliability of their findings.

**4. Statistical data analysis:**  The authors employed Pearson's product-moment correlation test to assess the association between wind speed (independent variable) and COVID-19 cases (dependent variable). However, it is important to note that Pearson's correlation test necessitates the use of normally distributed data. As the daily COVID-19 and wind speed data were not normally distributed, the utilization of Pearson's approach may prove problematic. Therefore, it is imperative that the authors examine the normality of the daily or weekly data used to inform their selection of the appropriate correlation test. In this context, Spearman's correlation test may be suitable since it does not rely on the approximate normality of the data. Furthermore, the authors categorized the strengths of the correlation between wind speed and COVID-19 into four groups: absence/weak relationship (r = 0.00–0.25), moderate (r = 0.26–0.50), strong (r = 0.51–0.75), and very strong/perfect (r = 0.76–1.00). It is necessary to elucidate the scientific rationale for this division and the guidelines employed. This will enhance the clarity and reliability of the authors' findings and recommendations.

Is the work clearly and accurately presented and does it cite the current literature?

Yes

If applicable, is the statistical analysis and its interpretation appropriate?

Partly

Are all the source data underlying the results available to ensure full reproducibility?

Yes

Is the study design appropriate and is the work technically sound?

Yes

Are the conclusions drawn adequately supported by the results?

Yes

Are sufficient details of methods and analysis provided to allow replication by others?

Partly

Reviewer Expertise:

Main research area is Biometeorology and Bioclimatology with current focus on weather and climate driven infectious diseases,  extreme heat and human health, air pollution and respiratory infections,  weather and climate extreme impacts, tropical climate variability, climate modelling and Urban Heat Island (UHI) warming,

I confirm that I have read this submission and believe that I have an appropriate level of expertise to confirm that it is of an acceptable scientific standard, however I have significant reservations, as outlined above.

# Response

**Published:** 2023-05-28

**Authors:** Dewi Susanna

**Affiliations:** Universitas Indonesia, Indonesia

**Dear Dr. Edmund I. Yamba**

 Department of Meteorology and Climate Science, Kwame Nkrumah University of Science and Technology (KNUST), Kumasi, Ghana

 Thank you very much for the valuable comments.

 Here, we tried to respond your comments as follow:

1. **The title vs the content presented: **

Based on your interpretation, it appears that the authors seek to establish the association between wind speed and COVID-19 cases in Jakarta, without delving into causality, as evidenced by their utilization of Pearson's correlation analysis. The use of the term "effect" in the title implies a notion of causality. Nevertheless, the research work put forth does not explicitly establish causality, but rather investigates the existence of a relationship through inferences. It is recommended that the authors reconsider the phrasing of the title to reflect the content of their study. For example, something like “The relationship between wind speed and COVID-19 infections in Jakarta”.

*Response:*

 Thank you for the proper title. The title of the article has been revised to " **The relationship between wind speed and COVID-19 infections in Jakarta"**

1. **Introduction/novelty: **

Despite the work's overall satisfactory and engaging presentation, the authors have not explicitly addressed the existing research gaps and their contributions to filling them. Previous studies have established the relationship between wind speed and the spread of COVID-19, which raises questions about the motivation behind the authors' replication of this work in Jakarta. Specifically, what were the compelling research questions that drove this inquiry and how were they answered? Hence, the originality or novelty of the work appears to be limited, and the authors should strive to enhance it. One suggestion for improvement could be to extend beyond the typical approach of establishing correlations between weather variables and COVID-19, which is prevalent in most existing literature. Merely identifying a correlation between two variables is insufficient to determine the causative relationship between them. Therefore, panel estimation techniques, for example, could be explored by the authors to investigate the causal aspect further.

*Responses:*

 The previous research gaps and the researcher's contribution in filling the previous research gaps (novelty) have been added in the introduction section:

 “In several countries, researchers have found that high wind speeds play a role in the increase of COVID-19 cases. ^7–12^ This is because SARS-CoV-2 can survive in the air, so wind speeds accelerate the spread of the virus by carrying droplets to various destinations. ^7^

 In the internal environment, higher wind speeds decrease the viral load indoors, which can prevent SARS outbreaks. ^14^ Other researchers have found that high wind speeds can actually suppress the spread of COVID-19 cases. ^15–17^

 This study considers some of the limitations of previous studies by examining the potential of wind speed in influencing the spread of cases based on regional characteristics ^7,9–12,15,17^ because environmental conditions between regions are different. ^18^ In addition, daily COVID-19 case data were converted into weekly cases to adjust for the incubation period of COVID-19 (5-6 days) and the uncertain time interval between the first day of infection and the day of sampling. ^19^

 As well as the potential spread of cases based on regional characteristics in DKI Jakarta, especially in coastal areas”.

1. **Data collection: **

*The authors conducted a study that involved the collection of daily wind speed and COVID-19 case data spanning March to September 2020, which was then converted into weekly data for 31 weeks. The rationale for the conversion remains unclear, and it would be beneficial if the authors could elucidate their reasons for doing so and how this approach facilitated more effective analysis. Additionally, it is essential to understand how gaps in the data were managed as the quality of the data used is fundamental to the reliability of the results obtained. The authors reported that the study was conducted in Jakarta province, consisting of 261 urban villages. However, it is not evident whether the data used were obtained from each of these villages and aggregated for the province. If this was the case, it would be useful to know whether data were available for each village. It is recommended that the authors provide more comprehensive details regarding the availability and quality of the data used in their analysis to enhance the reliability of their findings.*

*Responses:*

- The reason for converting daily covid-19 case data into weekly covid-19 case data has been added to the data collection section:

The conversion of daily cases to weekly cases is related to several considerations, such as the incubation period of COVID-19, which lasts for 5–6 days on average, and the uncertain time interval between the first day of infection and the day of sampling. ^19^

- An explanation of how to obtain covid-19 case data in 261 villages has been adjusted in the data collection section:

These data were obtained from case reports of hospitals, health centers, and laboratories recorded per urban village by the Jakarta Provincial Health Office and can be accessed on the Jakarta Provincial Health Office website ( https://corona.jakarta.go.id/id/data-pemantauan) and reports of two weather monitoring stations (Kemayoran station and Tanjung Priok station) in Jakarta, which can be accessed on the website of the Meteorology, Climatology, and Geophysics Agency ( https://dataonline.bmkg.go.id/akses_data ).

1. **Statistical data analysis: **

The authors employed Pearson's product-moment correlation test to assess the association between wind speed (independent variable) and COVID-19 cases (dependent variable). However, it is important to note that Pearson's correlation test necessitates the use of normally distributed data. As the daily COVID-19 and wind speed data were not normally distributed, the utilization of Pearson's approach may prove problematic. Therefore, it is imperative that the authors examine the normality of the daily or weekly data used to inform their selection of the appropriate correlation test. In this context, Spearman's correlation test may be suitable since it does not rely on the approximate normality of the data. Furthermore, the authors categorized the strengths of the correlation between wind speed and COVID-19 into four groups: absence/weak relationship (r = 0.00–0.25), moderate (r = 0.26–0.50), strong (r = 0.51–0.75), and very strong/perfect (r = 0.76–1.00). It is necessary to elucidate the scientific rationale for this division and the guidelines employed. This will enhance the clarity and reliability of the authors' findings and recommendations.

*Responses:*

- An explanation of the use of the pearson-product moment test has been added to the statistical data analysis section:

Before the correlation test is applied, the data normality test is conducted first. The results of the normality analysis show that the maximum wind speed variable is normally distributed, while the average wind speed and COVID-19 cases are not normally distributed. From these results, variables that are not normally distributed are then transformed by the square root (x) to obtain normality. This step is taken to fulfil the requirements for multiple linear regression analysis (normality assumption).

- The source of the correlation strength categorization has been provided and the reason for categorizing the correlation strength has been added:

In addition, the strengths of the association were qualitatively divided into four categories, where r = 0.00–0.25 was absence/weak relationship, r = 0.26–0.50 was moderate, r = 0.51–0.75 was strong and r = 0.76–1.00 was very strong/perfect. ^20^

 This categorization aims to determine how strongly the independent variables (maximum wind speed and average wind speed) correlate with the dependent variable (COVID-19 cases). It is also used to discuss the results of the analysis in the discussion section.

 Thank you for all the input so this article became more comprehensives and understandable  for the readers.
